# Clinical validation of controlled exposure to birch pollen in the Environmental Exposure Unit (EEU)

**DOI:** 10.1186/s13223-016-0156-7

**Published:** 2016-10-19

**Authors:** Anne K. Ellis, Mena Soliman, Lisa M. Steacy, Daniel E. Adams, Barnaby Hobsbawn, Terry J. B. Walker

**Affiliations:** 1 Division of Allergy & Immunology, Department of Medicine, Queen’s University, Kingston, ON Canada; 2 Allergy Research Unit, Kingston General Hospital, 76 Stuart Street, Kingston, ON K7L 2V7 Canada

**Keywords:** Allergic rhinitis, Environmental Exposure Unit, EEU, Environmental Exposure Chamber, Controlled allergen challenge facility, CACF, Birch pollen, Total nasal symptom score (TNSS), Peak nasal inspiratory flow (PNIF)

## Abstract

**Background:**

The Environmental Exposure Unit (EEU) in Kingston, Ontario, Canada is a controlled allergen challenge facility (CACF) that has been previously clinically validated for the use of ragweed and grass pollen in clinical studies. In this study we aim to validate the use of birch pollen to challenge allergic participants.

**Methods:**

A total of 59 volunteers were screened and 38 birch allergic participants and ten non-allergics completed the study, outside of tree pollen season. Participants had to have a minimum of 2-year history of allergic rhinoconjunctivitis during the typical tree pollen season and have a positive skin prick test to birch allergen ≥5 mm from the control. Qualified participants were exposed to birch (*Betula pendula*) pollen for 4 h in the EEU and recorded their symptoms of sneezing, rhinorrhea, nasal congestion, nasal itch which comprised the total nasal symptom score (TNSS), as well as itchy/watery eyes, red/burning eyes and itching of ears/palate/throat which along with the TNSS comprised the total rhinoconjunctival symptom score (TRSS) along with Peak Nasal Inspiratory Flow (PNIF) at baseline and at 30 min intervals for the duration of exposure, then hourly for up to 12 h from the start of exposure.

**Results:**

Allergic participants reported a gradual rise in TNSS and TRSS, reaching a mean and standard error of the mean of 7.08 ± 0.45 and 11.58 ± 0.93 respectively by 180 min from the start of exposure. Symptoms gradually declined to near baseline values following departing from the unit, reaching 1.9 and 2.7 by 450 min. Allergic participants reported significantly higher TNSS than non-allergics starting from 30 min (p < 0.01, two-way ANOVA with Bonferroni corrections), maintaining maximum significance from 60 to 300 min (p < 0.0001) and losing significance by 420 min. TRSS and PNIF followed similar trends as those seen with TNSS. Participants were phenotyped using previously published definitions using the TNSS into Early Phase Responders (EPR, 57.8 %), protracted EPR (pEPR, 39.5 %), and Dual Phase Responders (DPR, 2.7 %).

**Conclusions:**

The EEU can competently challenge birch allergic participants and achieve statistically significant changes in symptoms and nasal airflow, while such changes are not reported in non-allergic controls.

*Trial registration* NCT02351830 clinicaltrials.gov

## Background

Allergic rhinitis (AR) is an allergen-induced upper respiratory inflammatory disease characterized by hyperactive airway mucosa resulting in symptoms of rhinorrhea, sneezing, nasal pruritus, and congestion, with associated symptoms of red, itchy, watery eyes, itching of the palate and throat, and cough [1]. AR is widely recognized as the most common allergic condition, but detailed estimates of its actual prevalence are lacking. Canadian studies suggest the life-time prevalence of AR to be between 39 and 52 % [2]. AR is studied clinically using several types of allergen exposure models that mimic natural exposure under controlled conditions. On exposure to allergen, study participants experience very similar symptoms to those reported during natural exposure.

Studying AR in humans allows for better understanding of the pathophysiology of the disease and provides reliable methods for the evaluation of novel therapeutics in clinical trials. Nasal allergen challenge (NAC) is one method that involves the direct exposure of the nasal mucosa to the allergen of interest through a nasal spray device, paper discs containing the allergen or sometimes through direct pipetting [3]. The NAC method can be used in phase 2/3 clinical trials and has proven reliable in generating a variety of biological samples for possible mechanistic studies of novel medications [4, 5].

**Fig. 1:**
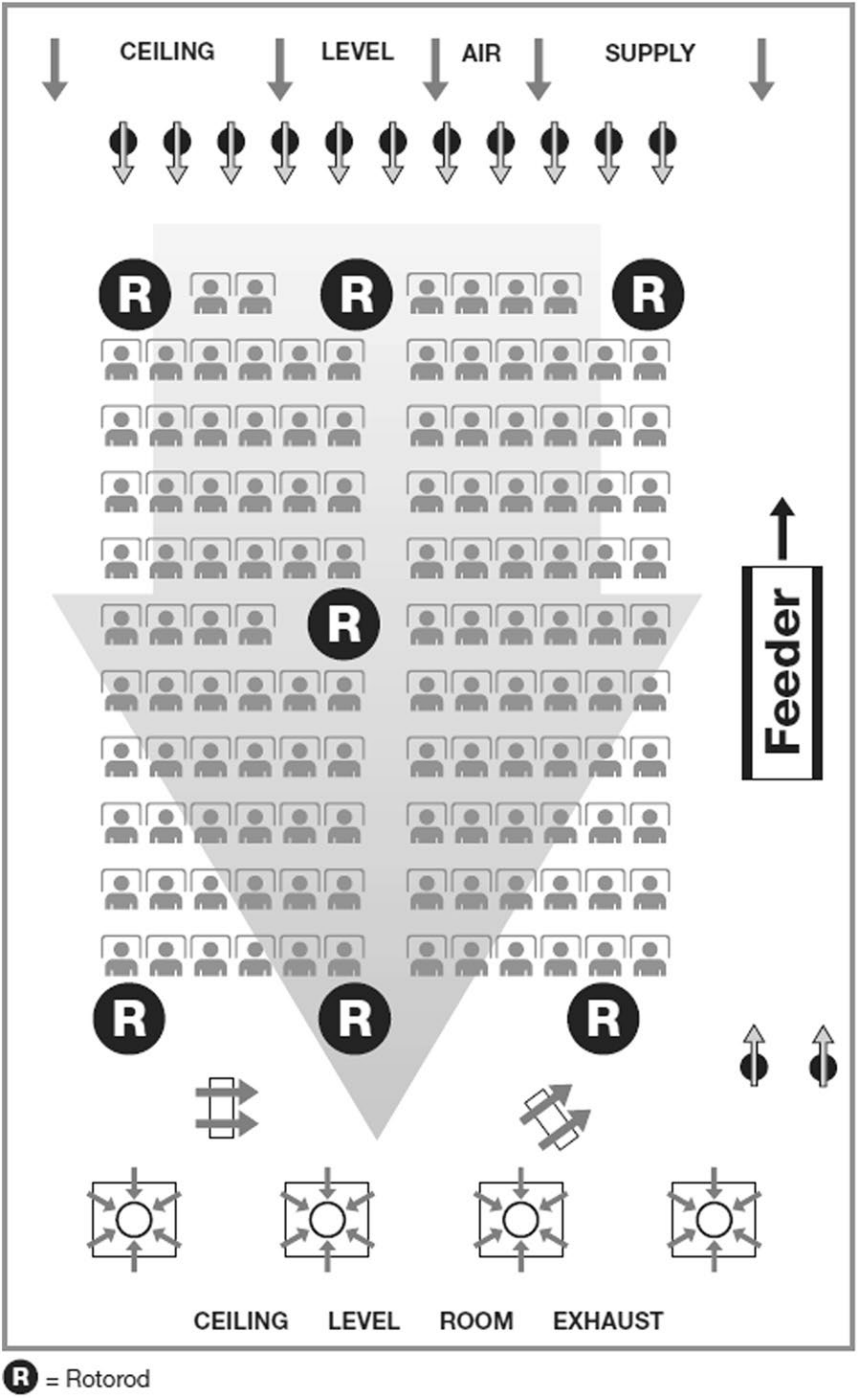
Schematic of the EEU. *Gray arrows* indicate direction of airflow. Adapted with permission from © Ellis et al. [18] licensee BioMed Central. 2015

Controlled allergen challenge facilities (CACFs), often otherwise referred to as “exposure units” or “exposure chambers” are specialized units that allow for the simultaneous exposure of many participants to controlled levels of allergen while also fully controlling the indoor air environment, including humidity, temperature, and CO_2_ levels; and additionally provide air filtration [6]. The Vienna Challenge Chamber (VCC) in Austria was the first multi-participant CACF to be developed in Europe [7]. The Environmental Exposure Unit (EEU) at Kingston General Hospital (KGH) in Kingston, ON, Canada, is the first such facility developed in North America and can accommodate up to 140 participants per exposure visit, and has been established to be a valuable tool in the development of several anti-allergic medications (Fig. 1) [8–12].

### The Environmental Exposure Unit (EEU) operation

The EEU allows for reliable allergen exposure of up to 140 participants at once while controlling the environment regardless of the outdoor weather conditions. A custom-engineered computer and laser-aided system controls the dispersion of a predetermined concentration of pollen from a single point of delivery. The pollen is propelled using directional fans over the seating area. Rotorod^®^ samplers (Sampling Technologies Inc, Minnetonka, Minnesota), placed in seven specific locations around the seating area, typically sample the pollen in the air every 30 min, allowing for the assessment of pollen concentration at each location (Fig. 1) [13–15]. Custom microcontroller regulated rotorods developed by the Allergy Research Unit team monitor and transmit their revolutions per minute (RPM) data, along with date and time stamp, wirelessly to the research data management system, and store information locally on secure digital storage. Sensors that monitor RPM, battery condition and rotorod spindle movement provide visual and audible feedback on-screen and through warning LED lights as well as a speaker in order to be able to alert of any potential operational issues. If required, environmental sensors can be fitted to the microcontroller to provide additional point source data of that particular location [15].

Through minor adjustments in the pollen dispersion system or directional fans during the exposure visit, pollen concentration remains consistent throughout the unit [16]. A special ventilation system effectively controls the indoor environment, providing 100 % filtered fresh outdoor air, while controlling humidity (adjusted between 40 and 60 %) and temperature (18–22 °C), and the CO_2_-laden air is exhausted outdoors [8]. The system is capable of replacing the volume of the room with filtered fresh air once every 12 min, while maintaining the pre-set temperature and humidity.

Due to the geographical location of the EEU and the high prevalence of ragweed allergy in Kingston, ON, ragweed has been extensively used as the allergen of choice during clinical trials [10, 17, 18]. Recently, there has been a growing need to validate and use other allergens for the evaluation of allergen specific immunotherapies in facilities like the EEU, as opposed to previous studies of anti-histamines and intranasal corticosteroids, where the specific allergen used is not as critical [10, 17–19]. Since different pollens have varying physical properties such as weight and air dynamics, which would affect their suspension in the air and ultimately the concentration, there is a need to evaluate the distribution and clinical effects of different pollens within the EEU before incorporating their use into future clinical studies. The use of grass pollen was most recently evaluated in the EEU, providing clinical validation of its use, and determining the pollen concentration and distribution requirements needed to reach predetermined symptom scores [16].

**Fig. 2:**
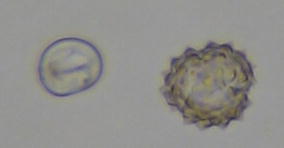
Topographic features of birch and ragweed pollen on microscopic examination (not to scale). Birch pollen (*left*) have smoother surfaces with three pores compared to ragweed pollen which have spiky and barbed surfaces that causes the pollen grain to clump together. Both pollen are of comparable size, 20–22 microns in the case of birch pollen and 18–20 microns for ragweed pollen

Ragweed pollen has a barbed and spiky surface giving it a “sticky” property and the clumping of pollen grains together may cause it to remain aloft during increased air current velocities (Fig. 2). Birch pollen, which has a comparable particle size to ragweed (20–22 microns compared to 18–20 microns for ragweed), would be expected to share similar air suspension characteristics. Having three raised pores on its surface, birch pollen may be able to remain suspended in the air for longer periods before “falling out” of air currents and coming to rest, similar to the effect of spikes on ragweed pollen. A birch pollen concentration of 3500 ± 500 grains was targeted for this study, similar to previous ragweed studies [8]. Preliminary studies in the EEU while fully setup, but without human participants, have confirmed the capability of the system equipment to release, disperse and maintain birch pollen concentrations [20].

We aimed to clinically evaluate the use of European White Birch pollen (*Betula pendula*) to challenge birch allergic participants and establish the change in symptom scores and nasal air flow at different time points throughout and following pollen exposure.

## Methods

### Participants

Individuals on file from previous enrolment in studies with the Allergy Research Unit of KGH were approached to participate in this study. Inclusion criteria for the allergic population included males or females between the ages of 18–65 years, a minimum 2 year documented history of allergic rhinoconjunctivitis symptoms during the typical tree pollen season (mid-April to early June), and have a positive skin prick test (SPT) to birch allergen at screening with a wheal diameter ≥5 mm than the negative control. Participants had to be willing and able to provide written informed consent and comply with study requirements. Additionally, sexually active women of childbearing potential were asked to use a medically acceptable method of birth control, and produce a negative urine pregnancy test at screening. Non-allergic participants had to meet the same criteria except for the history of AR and were required to have negative skin test responses to a panel of common environmental allergens, including birch.

Exclusion criteria for all participants included having an upper respiratory tract infection within 1 week of pollen exposure, participants with asthma requiring the use of a short-acting beta agonist greater than twice a week, or anyone with a history of birch-pollen induced asthma, regardless of severity, or a history of any disease that in the judgement of the investigator would impact on the participant’s safety. Similarly, participants were excluded if they had a history of positive test results for Hepatitis B, Hepatitis C, HIV, or tuberculosis (other than due to vaccination), or significant history of drug or alcohol abuse or other clinically relevant abnormalities on physical exam. Other exclusion criteria were females who were pregnant, actively trying to become pregnant, or currently lactating. Participants were also asked to observe washout periods for medications listed in Table 1.

**Table 1:** Washout periods for medications

Medication	Duration of washout prior to the pollen exposure visit
Beta-blockers, alpha-adrenoceptor blockers, currently receiving allergen immunotherapy	Not permitted
Topical alpha-adrenergic agonists	48 h
H1 receptor antagonists	7 days
Topical corticosteroids^a^	7 days
Anticholinergics	7 days
Intranasal or inhaled corticosteroids	14 days
Intranasal or inhaled cromolyn	14 days
Tricyclic antidepressants and monoamine oxidase inhibitors	14 days
Leukotriene inhibitors	14 days
Systemic corticosteroids (oral)	30 days
Depot corticosteroids	60 days

Participants were asked to follow the washout periods of the medications below before the pollen exposure visit

^a^Hydrocortisone ≤1 % used on <10 % body surface area was permitted throughout the study

The study was reviewed and ethics clearance granted by the Queen’s University and Affiliated Teaching Hospitals Research Ethics Board (REB), and was registered at clinicaltrials.gov (NCT02351830).

### Study design

The study was conducted outside of pollen season (February 2015). At the screening visit, participants provided written informed consent and had their vital signs, height and weight measured. A medical history was taken and physical examination, including nasal examination, was conducted. SPT was performed on the volar surface of the participant’s forearm for the following allergens: Birch, timothy grass, rye grass, short ragweed, tree mix, dog, cat, dust mite (*D. pteronyssinus, D. farinae*), and *Alternaria* mould.

Qualified participants were invited back to the EEU for one 4 h birch pollen exposure session. Before the exposure, the inclusion and exclusion criteria were reviewed and an infectious disease questionnaire was completed by the participants to ensure they were in good health. Women of childbearing potential were required to have a negative pregnancy test.

Participants were seated inside the EEU and birch pollen (Greer, NC) was delivered and maintained at a concentration of 3500 ± 500 grains. The pollen concentration was determined every 30 min using seven Rotorod^®^ samplers placed at specific locations and the pollen emission rate was then modified based on the Rotorod^®^ counts to maintain equal distribution of the pollen throughout the facility. Other environmental factors were controlled during the exposure period as described earlier.

Participants used either paper diary cards or electronic tablets [21] to record their total nasal symptom score (TNSS) at baseline and at 30 min intervals for the duration of the exposure, then hourly up to 12 h from the start of pollen exposure. Participant symptoms were captured using both paper diary cards and as electronic patient-reported outcomes (ePRO) recorded on tablets. Both means to capture the participants’ symptoms resulted in data being stored in our validated Clinical Trial Data Management System. All participants recorded their symptoms from hours 4–12 on paper diary cards and mailed them back to the site upon completion. At each time point participants graded their symptoms on a scale from 0 to 3, including sneezing, runny nose, itchy nose, and congestion, for a total out of 12 (Table 2). Participants also recorded ratings of symptom severity for itchy ears/palate/throat, itchy/gritty eyes, red/burning eyes, and teary eyes, and these scores, in addition to the TNSS, comprised the Total Rhinoconjunctivitis Symptom Score (TRSS) for a maximum score of 24. Participants were trained to measure peak nasal inspiratory flow (PNIF) using a facial mask and meter (InCheck, Clement Clarke International Ltd, Essex, UK), taking three measurements at each time point. The greatest of the three measurements was used as the final measure of air flow.

**Table 2:** Symptom score definitions

Score	Definition
0 = none	Symptom is completely absent
1 = mild	Symptom is present but minimal awareness, easily tolerated
2 = moderate	Awareness of symptoms, bothersome, but tolerable and not interfering with daily activities
3 = severe	Definite awareness of symptoms, difficult to tolerate, interferes with activities; and/or desires treatment

Participants graded each symptom on a 3-point Likert scale (0–3) every 30 min. The total score was added up for a total out of 12 (TNSS) and 24 (TRSS)

Biological samples were collected during this study, including nasal brushing for sampling epithelial cells and blood samples for PAX gene analysis and CBC differentials. The results from these analyses will be reported in future submissions.

### Statistical analysis

GraphPad Prism 6.0 (San Diego, CA, USA) was used for the statistical analysis of the data. TNSS, TRSS, and PNIF data from allergic and non-allergic participants were compared using two-way repeated measures ANOVA with Bonferroni’s correction. Comparisons of scores at different points to baseline was completed using one-way repeated measures ANOVA with Tukey’s correction. The percentage reduction in PNIF at each time point compared to baseline was used to compare allergic and non-allergic groups.

## Results

**Fig. 3:**
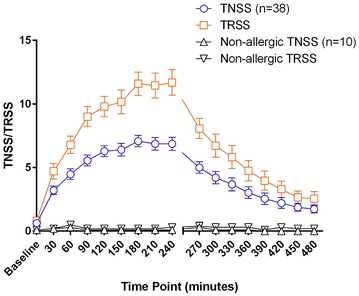
Increase in TNSS and TRSS reported by birch allergic participants during the EEU challenge visit. Birch allergic participants reported a gradual increase in total nasal symptom scores (TNSS) and total rhinoconjunctivitis symptom scores (TRSS), reaching a maximum of 7 and 11.6 respectively at 180 min, and sustaining this level until the end of the exposure period at 240 min. Symptoms gradually returned to near baseline values following the end of exposure. Non-allergic participants did not report such changes and had no statistically significant increase in scores from baseline

**Fig. 4:**
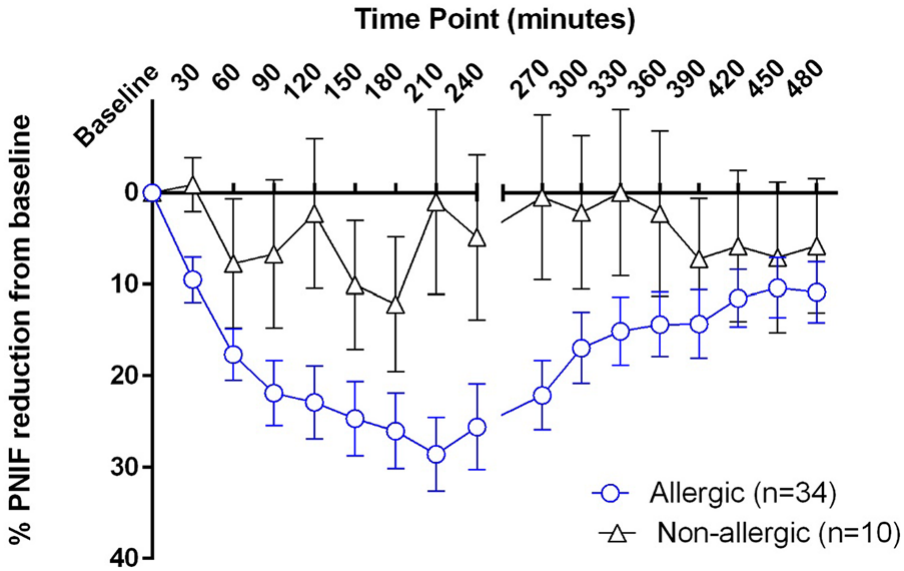
Decrease in PNIF reported by birch allergic and non-allergic participants during the EEU challenge visit. Birch allergic participants experienced a reduction in their peak nasal inspiratory flow (PNIF) compared to baseline, which was sustained for the duration of the exposure period (up to 240 min time point) and peaking at a mean reduction of 28.6 % at 210 min. The PNIF gradually decreased following the challenge but did not return to baseline values by the 480 min time point. Non-allergic participants reported wide variation in their PNIF but with no statistically significant change from baseline values throughout the duration of the study (one-way repeated measures ANOVA with Tukey’s correction). NB: four participants did not complete the PNIF section of the diary cards they took home (270 min time point to 480 min) and were excluded from this analysis

Fifty-nine volunteers were screened for enrollment; a total of 38 birch allergic and ten non-allergic participants completed the study; one allergic participant failed to return all the take-home diary cards and was excluded from the analysis. A further four participants neglected to record PNIF values on the diary cards completed after leaving the EEU and were thus excluded from the PNIF analysis. Prior to pollen exposure, TNSS, TRSS and PNIF recordings were similar for both allergic and non-allergic groups with no statistical difference (Figs. 3, 4). Allergic participants experienced a gradual rise in TNSS and TRSS, reaching a mean score and standard error of the mean of 7.08 ± 0.45 and 11.58 ± 0.93 respectively at 180 min from the start of exposure, and maintained this level until the end of the visit at 240 min. TNSS and TRSS gradually declined after leaving the EEU, up to 450 min from the start of the study at which point both symptom scores reached a nadir of 1.9 ± 0.32 and 2.7 ± 0.49 respectively (Fig. 3).

Non-allergic participants reported no change in their symptoms over the entire duration of the study. Allergic participants reported statistically higher TNSS than non-allergic participants at most time points, starting from the 30 min time point (p < 0.01), reaching and maintaining maximum significance from 60 to 300 min (p < 0.0001), declining gradually until 420 min at which statistical significance was lost (Table 3). TRSS followed a similar trend (Fig. 3; Table 3).

**Table 3:** Statistically significant change in TNSS, TRSS, and PNIF when comparing allergic and non-allergic participants and comparing baseline to each time point for allergic participants

	Time point (minutes)	Statistical significance (p)
TNSS: allergic vs non-allergic participants	Baseline	NS
	30	<0.01
	60	<0.0001
	90	<0.0001
	120	<0.0001
	150	<0.0001
	180	<0.0001
	210	<0.0001
	240	<0.0001
	270	<0.0001
	300	<0.0001
	330	<0.001
	360	<0.05
	390	<0.05
	420	NS
	450	NS
	480	NS
TRSS: allergic vs non-allergic participants	Baseline	NS
	30	NS
	60	<0.001
	90	<0.0001
	120	<0.0001
	150	<0.0001
	180	<0.0001
	210	<0.0001
	240	<0.0001
	270	<0.0001
	300	<0.001
	330	<0.01
	360	NS
	390	NS
	420	NS
	450	NS
	480	NS

	Time point (minutes) vs baseline	Statistical significance (p)
TNSS: allergic participants, each time point vs baseline	30	<0.0001
	60	<0.0001
	90	<0.0001
	120	<0.0001
	150	<0.0001
	180	<0.0001
	210	<0.0001
	240	<0.0001
	270	<0.0001
	300	<0.0001
	330	<0.0001
	360	<0.001
	390	<0.01
	420	<0.05
	450	<0.01
	480	<0.01
PNIF: Allergic participants, each time point vs baseline	30	<0.05
	60	<0.0001
	90	<0.0001
	120	<0.001
	150	<0.0001
	180	<0.0001
	210	<0.0001
	240	<0.001
	270	<0.001
	300	<0.01
	330	<0.05
	360	<0.05
	390	<0.05
	420	NS
	450	NS
	480	NS

Allergic participants reported statistically significant increase in TNSS and TRSS and reduction in PNIF compared to non-allergic participants at most time points. Within the allergic group, changes in TNSS and PNIF, following allergen exposure, was significantly different from baseline measurements

Within the allergic participants, the rapid increase in TNSS was statistically significant compared to their baseline measurements at all time points (Table 3). A similar trend was observed with TRSS while no such significant change was reported by non-allergic participants.

PNIF recorded by allergic participants followed a similar trend to TNSS and TRSS, though nasal air flow never returned to baseline values by the end of the study period (Fig. 4). While non-allergic participants experienced no statistically significant change in PNIF compared to baseline, birch allergic participants reported a significant reduction in their PNIF at most time points (Table 3).

The percentage change in PNIF from baseline is another method used to analyze nasal air flow, providing further comparison between allergic and non-allergic participants. Non-allergic participants recorded greater variability in PNIF Due to this variability, it was difficult to compare allergic to non-allergic participants, though the difference in pattern was visually apparent.

**Fig. 5:**
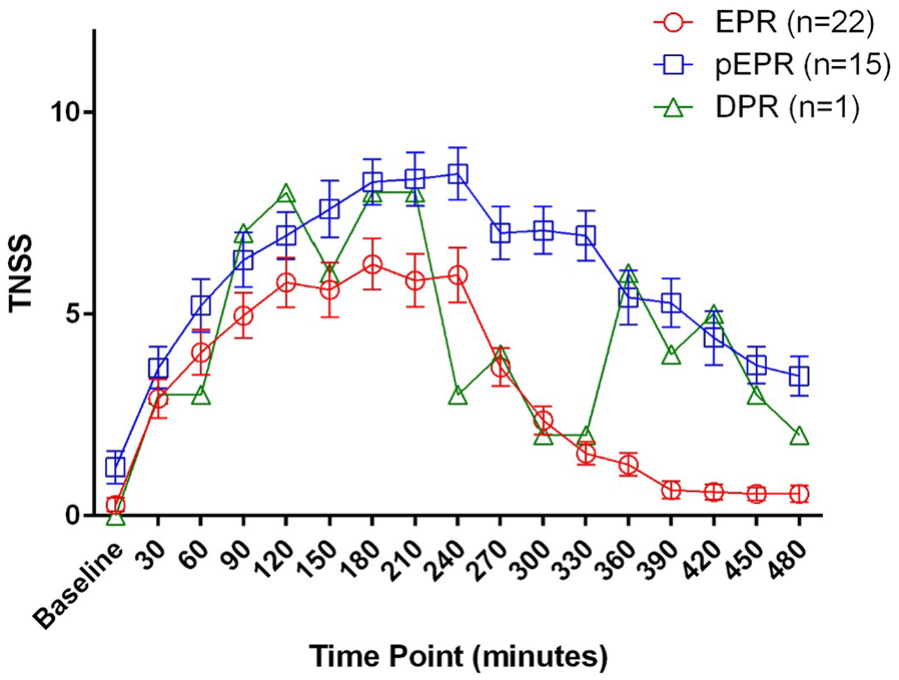
Birch allergic participants were phenotyped into EPR, pEPR and DPR according to their TNSS responses. Participants who experienced an initial rise in their TNSS followed by a decrease of 50 % or more by the 6th or 7th hour, with no second rise in symptoms, were defined as early phase responders (EPR). Participants who reported a second rise in symptoms following the 50 % decline were classified as a dual phase responders (DPR), while those who did not report a 50 % reduction in symptoms but rather a relative maintenance of symptoms were defined as protracted EPR (pEPR)

Participants were phenotyped according to their TNSS pattern using previously defined and published definitions (Fig. 5) [22]. Twenty-two participants (57.8 %) experienced a gradual rise in their TNSS followed by a reduction of 50 % from the peak score by the 6th or 7th hour and were classified as Early Phase Responders (EPR). Fifteen participants (39.5 %) reported a similar gradual rise in symptoms but did not experience a reduction of 50 % in symptoms by the 6th or 7th hour, and were classified as having a protracted EPR (pEPR). One sole participant in this study met the criteria for a Dual Phase Responder (DPR), in that they experienced a 50 % reduction in TNSS by the 6th or 7th hour followed by an increase of at least two points thereafter.

## Discussion

Birch allergic participants were clearly able to experience symptoms of AR due to controlled birch pollen challenge in the EEU outside of tree pollen season. The symptoms experienced were statistically higher than that experienced by non-allergic participants, the majority of whom experienced no symptoms at all. The single 4-h session was also able to elicit eye-related symptoms of allergic conjunctivitis. Accompanying the development of rhinitis symptoms, participants experience a significant reduction in nasal air flow.

The results from the study suggest that the birch pollen concentration of 3500 ± 500 grains was an adequate concentration to target and produced the intended results in a period of time mirroring a ragweed exposure. This may be attributed to similarities in topography and size between ragweed and birch pollen, and as such the target concentration used for ragweed studies was also appropriate for this study using birch pollen.

Participants in this study demonstrated a variety of AR phenotypes, allowing for the future study of the effect of novel therapies in each of EPR, pEPR and DPR. It was noteworthy that challenge with birch pollen appears to be associated with a lower rate of DPR generation than seen previously following a ragweed allergen challenge [22]. Overall, however, the distribution of participants demonstrating the different phenotypes is similar to previously published data using ragweed allergen to challenge participants at the EEU [22].

One observed difference between the current study and our previous investigation involving a ragweed challenge is the slightly lower peak TNSS in the birch evaluation; a mean peak TNSS of 9.2 was observed in the ragweed study [17] compared to 7.1 in the birch study. The study that validated the EEU for grass pollen challenges also yielded slightly different responses to our current evaluation. This study was of a slightly different design, and challenged participants over two consecutive days for 3 h to evaluate effects of repeated exposures [16]. As a result of this “priming” effect, a mean peak TNSS of 9.2 was reached on the second day. The single birch pollen exposure session in this study, while resulting in a lower mean peak TNSS (7.1) was still enough to achieve and exceed the typical target TNSS of 6 often used for CACF type studies [23–26]. Higher scores might be achieved, if needed, by adding priming sessions to “re-awaken” the allergic reaction to pollen when studying AR outside of the relevant pollen season [6, 27].

## Conclusions

The EEU provides a controlled environment for effectively studying birch induced AR outside of the pollen season, adding to the previous toolkit of ragweed and grass pollen challenge expertise. Such capability allows for the testing of allergen specific novel immunotherapies in a controlled environment while accommodating up to 140 participants per study session.

## Abbreviations

CACF: controlled allergen challenge facility
EEU: Environmental Exposure Unit
PNIF: peak nasal inspiratory flow
SPT: skin prick test
TNSS: total nasal symptom score
TRSS: total rhinoconjunctival symptom score
